# An Evaluation of Replacing Soybean Meal with Proteolytic Soybean Meal in Low-Fish-Meal Diet on Growth Performance, Expression of Immune-Related Genes, and Resistance against *Vibrio alginolyticus* in White Shrimp (*Litopenaeus vannamei)*

**DOI:** 10.1155/2022/8384917

**Published:** 2022-08-31

**Authors:** Weilong Wang, Detian Jiang, Ganfeng Yi, Xuxiong Huang

**Affiliations:** ^1^Centre for Research on Environmental Ecology and Fish Nutrition of the Ministry of Agriculture and Rural Affairs, Shanghai Ocean University, China; ^2^Shanghai Collaborative Innovation Center for Cultivating Elite Breeds and Green-Culture of Aquaculture Animals, Shanghai 201306, China; ^3^National Demonstration Center for Experimental Fisheries Science Education, Shanghai Ocean University, China; ^4^Beijing Dabeinong Technology Group Co., Ltd., Beijing 100080, China

## Abstract

A 56-day feeding trial was conducted to investigate the effects of dietary proteolytic soybean meal (PSM) on growth performance, immune-related genes, and resistance against *Vibrio alginolyticus* in *Litopenaeus vannamei*. Six dietary levels of PSM (0, 35, 45, 55, and 65 g/kg) were added to a basal diet. The results showed that juveniles fed more than 45 g/kg PSM exhibited significantly (*P* < 0.05) promoted growth performance compared to the control. Furthermore, all PSM supplemented treatments showed significantly better performances in terms of feed conversion ratio (FCR), the protein efficiency ratio (PER), and the protein deposition ratio (PDR). Corresponding to the performances on growth and nutrient utilization, a significantly higher protease activity in hepatopancreas was also obtained in all PSM incorporations. Also, the activities of immune-related enzymes such as superoxide dismutase (SOD) and lysozyme in serum were significantly (*P* < 0.05) elevated in shrimp fed with PSM. Notably, shrimp fed with the 65 g/kg PSM supplemented diet showed significantly (*P* < 0.05) lower cumulative mortality compared to the control after challenging with *Vibrio alginolyticus* injection at 72 h. PSM supplementation significantly (*P* < 0.05) upregulated expression levels of immune deficiency (IMD) and Toll-like receptor 2 mRNA in shrimp gill tissues directly or indirectly reflected their activation effect in shrimp innate immune response. In conclusion, the current study proved that partial replacement of soybean meal with PSM could result in better growth and immune status of *L. vannamei*.

## 1. Introduction

Since 1980, shrimp catches have declined linearly with the rapid development of aquaculture [1]. Intensive farming systems combined with artificial aquafeed represent the only viable way of bridging the gap between the increasing demand and supply of this high-value seafood [2]. With the enormous expansion of aquaculture activities, the demand for aquafeed also increased [3]. The utilization of fish meal in commercial feeds is very common and varies from 25 to 50% depending on the species. Fish meal is the preferred protein source due to its unique nutritional characteristics, which approximate almost precisely the nutritional protein requirements of cultured shrimp [4]. Moreover, apart from providing essential amino acids (EAA), fish meal is a good source of essential fatty acids, digestible energy, macro and trace minerals, vitamins, attractants, and many other finite micronutrients [5]. Therefore, it has been predicted that the future demand for the fish meal would increase day by day within the compound for the aquafeed manufacturing industry [6]. The constant annual output in contrast to the continuously increasing demand for the fish meal raised its price and the cost of aquafeed [7]. There, reducing the entire dependence upon fish meal and finding out cost-effective alternative protein sources is the prerequisite for the profitable and sustainable aquaculture industry.

Besides the full availability and competitive price, a viable candidate must possess specific nutritional characteristics, such as having a relatively high protein content, favorable amino acid, high nutrient digestibility, reasonable palatability, and low levels of nonsoluble carbohydrates and antinutrients [8]. Thus, various plant proteins, including soybean meal, canola meal, peanut meal, and corn protein used in aquafeeds, have been considered an essential requirement for the future development of aquaculture around the world [9]. Soybean products are the most utilized plant protein as an alternative to fish meal in the diets of fish and shrimp because of their high protein contents, digestibility, and price [10]. To date, many researchers have conducted numerous studies on the replacement of fish meal in the aquafeed by using different types of soybean meal. It was found that 37-62% of the fish meal could be replaced by soybean meal in *L. vannamei* [11], *Fenneropenaeus indicus* [12], *Portunus pelagicus* [7], *Marsupenaeus japonicus* [4], and *Penaeus monodon* [13]. In comparison with fish meal, imbalance amino acids (especially lysine, methionine, and threonine), lower availability of phosphorus and cationic minerals, higher concentrations of indigestible carbohydrates, poorer palatability, and some unknown antinutritional factors in soybean meal made it unsuitable to completely replace fish meal due to compromising the growth performance and nutrient utilization of crustaceans [5]. Thus, commonly supplementing certain amino acids, phytase, and minerals could partially compensate for the nutritional deficiencies in soybean meal [14]. Previous studies showed that fermentation and proteolysis increased crude protein content and decreased crude fiber, antinutritional factors, and toxins [15]. Compared with conventional soybean meal, proteolytic soybean meal (PSM) contains a large number of small peptides, which performed better physical and chemical properties, and further increased its digestibility [10].

*L. vannamei* has become one of the essential crustacean species in China, due to its substantial economic value, high growth rate, and strong tolerance to a wide range of salinity and temperature [16]. Continued growth and intensification of aquaculture production depend upon sustainable protein sources to replace fish meal in aquafeeds. To the best of our knowledge, few studies have been conducted on immune and anti-stress performance after feeding PSM. Therefore, investigating critical factors such as growth performance, survival rate, body composition, various typical immune-related gene expression, and resistance against *Vibrio alginolyticus* in the current study would clarify the effects of dietary supplementation of PSM in a low-fish-meal diet.

## 2. Materials and Methods

### 2.1. Diet Preparation

Five isonitrogenous and isolipidic (about 40% crude protein and 13% crude lipid) experimental diets were formulated with different levels (0, 35, 45, 55, and 65 g/kg) of PSM (Table 1). PSM, a complex enzyme-treated soybean meal (about 48% crude protein and 0.9% crude lipid), was subjected to a proprietary process according to the specification of the producer. The molecular mass distribution of water-soluble nitrogen in enzymatic hydrolyzed soybean meal was measured before using (including >5000 ku 10.21%, 5000-3000 ku 4.43%, 3000-2000 ku 4.22%, 2000-1000 ku 8.32%, 1000-500 ku 11.49%, 500-180 ku 16.63%, and <180 ku 44.71%). The basal diet (control) was formulated with 50 g/kg brown fish meal inclusion. Corn protein powder, soybean meal, and peanut meal were used as major protein sources. Methionine was supplemented to simulate the amino acid profile of shrimp tissue. Fish oil, squid paste, and soybean lecithin were used as the major lipid and phospholipid sources. Vitamins, minerals, and other ingredients were added to meet the growth requirements for juvenile shrimp. The diet-making process started with mixing all dry ingredients thoroughly with a mixer (B10, Rudong, China) for 15 minutes. Lipid ingredients and fat-soluble components were also premixed, then added to the dry ingredients with continuous mixing. Water (about 30%) was subsequently added and carefully mixed for another 15 min. Finally, the blend was transferred into a single-screwed mincer to produce pellets of 1.5 mm diameter. The pellets were dried until moisture content was reduced to 10% and stored at -20°C until use. The biochemical analysis of the diets was shown below in Table 1.

### 2.2. Feeding Management

Juveniles used in the current study were purchased from Chunliang Aquaculture Cooperatives (Shanghai, China). Before the start of the feeding trial, juveniles were maintained in an indoor cement pool and fed with a commercial diet (40% crude protein and 8% crude lipid) for two weeks. After acclimatizing to laboratory conditions, 750 healthy shrimp of similar size (initial wet weight of approximately 0.23 g) were randomly selected and allocated to PVC net cages (Length = 1 m, Width = 1 m, and Height = 1.2 m) fitted inside a cement pond (Length = 10 m, Width = 5 m, and Height = 1.5 m) corresponding to 50 shrimp/cage for five dietary treatments in triplicate. All juveniles were fed four times every day (5:30, 10:30, 16:30, and 22:00) at a daily ration of 6-10% of body weight for eight weeks. The daily amount of the diets were adjusted every week by estimating the total weight of shrimp in each cage. Three hours after feeding, the uneaten feed was collected, while fecal matters were siphoned from the cages. All uneaten food was freeze-dried to calculate feed intake and feed efficiency ratio. During the feeding trial, all pools were provided with continuous aeration. Water quality parameters, including ammonia nitrogen, temperature, pH, salinity, and dissolved oxygen, were maintained at <0.05 mg/L, 30-32°C, 8.0-8.5, 1-2‰, and >5 mg/L, respectively.

### 2.3. Sample Collection and Biochemical Analysis

At the end of the feeding trial, all juveniles were fasted for 24 h before final sampling to empty their guts. The total number of survivors and the individual body weight of shrimp from each cage were measured. Crude protein, crude lipid, moisture, and ash contents of experimental diets and shrimp body samples were measured per the standard methods of the Association of Official Analytical Chemists [17]. Briefly, moisture was determined by oven-drying at 105°C to a constant weight. Ash by incinerating in a muffle furnace at 550°C for six hours. Crude protein by the Kjeldahl method (2300-Autoanalyzer, Foss Tecator, Sweden) was analyzed. Crude lipids by the Soxhlet extraction method was analyzed.

Five shrimp from each cage were obtained for immunological and antioxidant capacity analysis. Hemolymph was collected from the shrimp ventral sinus using a 1 mL sterile syringe. The serum was collected by centrifuging hemolymph samples at 10, 000 rpm for 10 min. The biochemical indices of the serum were determined within 24 h of storage at 4°C.

Digestive enzyme activities were analyzed using the hepatopancreas excised from five shrimp for each cage in 1 h after the final sampling to guarantee maximum activity. The hepatopancreas was collected, weighed, and homogenized with sterilized shrimp saline at a ratio of 1 : 9 (*W*/*V*). After centrifugation, the supernatant fluid was collected for digestive enzymes analysis. Amylase, protease, and lipase activities were determined using kits according to the manufacturer's instructions (Nanjing Jiancheng Bioengineering Institute, China).

### 2.4. Susceptibility of Shrimp to *Vibrio alginolyticus*

A pathogenic *V. alginolyticus* strain was isolated from diseased *L. vannamei* hemolymph sample on thiosulfate citrate bile salts sucrose (TCBS) agar culture medium plates to obtain virulent clones for the formal infection test per Yan et al. [16]. A susceptible test of shrimp to *V. alginolyticus* was conducted at the end of the eight-week feeding trial. Thirty juveniles from each treatment injected with 25 *μ*L activated *V. alginolyticus* (6.25 × 10^7^ CFU/mL) were placed in a (50 cm × 30 cm × 80 cm) cage suspended in a cement pool with continuous aeration, a water temperature of 29-30°C, and dissolved oxygen >6 mg/L in triplicates. The control group was injected with equivalent physiological shrimp saline. The water was renewed daily, and the experiment lasted for four days.

### 2.5. Total RNA Extraction, Reverse Transcription, and Quantitative Real-Time PCR

Total RNA was extracted from the gill tissues of shrimp within similar sizes and injected 25 *μ*L inactivated *V. alginolyticus* (1.25 × 10^7^ CFU/mL) from each treatment according to the manufacturer's instructions for Total RNA Extraction Kits (Tiangen, China). Isolated RNA quantity and quality were determined using a NanoDrop 2000 (Thermo, US) spectrophotometer and a 1% agarose gel electrophoresis. The extracted total RNA was transferred to cDNA by PrimeScript™ RT reagent Kits (Takara, Japan). Gene-specific primers were designed using Primer Premier 5.0 software based on the cDNA sequences in GenBank (Toll-like receptor 2 (GenBank: DQ923424.1) and immune deficiency (IMD) (GenBank: FJ592176.1)) (Table 2). The procedure of amplification and quantitative PCR were performed per the method of Yan et al. [16]. The relative expression levels of target genes were calculated using the 2^-*ΔΔ*Ct^ method described by [18].

### 2.6. Calculations

Specific growth rate (SGR, %/day) = [(Ln final body weight − Ln initial body weight)/duration] × 100, 

Feed conversion ratio (FCR) = dry weight of diet consumed (g)/live weight gain (g), 

Survival (%) = (final number of shrimp/initial number of shrimp) × 100, 

Protein efficiency ratio (PER, %) = live weight gain (g)/total protein intake (g) × 100, 

Protein deposition ratio (PDR, %) = 100 × (final body weight × final body protein − initial body weight × initial body protein)/(dry weight of diet consumed × protein of diet).

### 2.7. Statistical Analysis

The statistical analysis was performed by using variance (SPSS 20.0) after exploring the normality and homogeneity of the data. Results are presented as means ± S.E.M. (*n* = 3). One-way analysis of variance (ANOVA) was employed to test the mean effect of dietary manipulation. When there were significant differences (*P* < 0.05), the group means were further compared to the control by Duncan's new multiple range test.

### 2.8. Ethical Statement

The present experimental procedures were carried out in strict accordance with the recommendations in the ethical guidelines of EU Directive 2010/63/EU for animal experiments.

## 3. Results

### 3.1. Growth Performances and Feed Utilization

The results of the growth and feed utilization of the juvenile *L. vannamei* fed with different levels of PSM are shown in Table 3. All treatments had similar survival of around 97%, with no significant differences among the experimental groups. Dietary PSM levels significantly affected the growth and feed utilization (*P* < 0.05). FBW and SGR significantly increased with the increase in dietary PSM levels from 0 to 45 g/kg. However, there was no significant difference with the further increase in dietary PSM levels. The juveniles fed with PSM-supplemented diet showed significantly better performance on FCR, PER, and PDR compared to the control. However, no significant difference (*P* > 0.05) was detected among all the PSM supplementation treatments.

### 3.2. Biochemical Analysis of Shrimp Muscle

Effects of dietary PSM levels on the proximate composition of the muscle of shrimp after the feeding trial are presented in Table 4. The crude protein in shrimp muscle increased with the increase in dietary levels of PSM. Furthermore, compared to the control, 55 g/kg PSM supplemented treatments showed significantly higher (*P* < 0.05) values. However, moisture, ash, and total lipid content in the muscle did not show any statistical differences among all the treatments.

### 3.3. Digestive Enzyme Activities

The digestive enzymes activities of *L. vannamei* fed with the experimental diet for 56 days are shown in Table 5. The dietary PSM level significantly influenced the amylase, lipase, and protease activities in hepatopancreas. The significantly higher amylase was observed in 4.5 and 65 g/kg PSM-supplemented treatments compared to the control. Only a 35 g/kg dietary level showed significantly differ (*P* < 0.05) from the control in lipase activity. However, significantly higher protease activity values were obtained in all PSM supplemented treatments.

### 3.4. Nonspecific Immunity Parameters

The effects of dietary PSM levels on immunological and antioxidant capacity are presented in Table 6. Lysozyme, SOD, and MDA in hemolymph were significantly affected by dietary PSM levels (*P* <0.05). All PSM supplemented treatments showed significantly higher (*P* < 0.05) values of lysozyme and SOD compared to the control. Shrimp fed the diet containing 65 g/kg PSM showed significantly lower (*P* < 0.05) MDA than the control, whereas no significant difference was detected among all PSM dietary treatments.

### 3.5. Cumulative Mortality of *L. vannamei* after Challenging with *V. alginolyticus*

**Figure 1:**
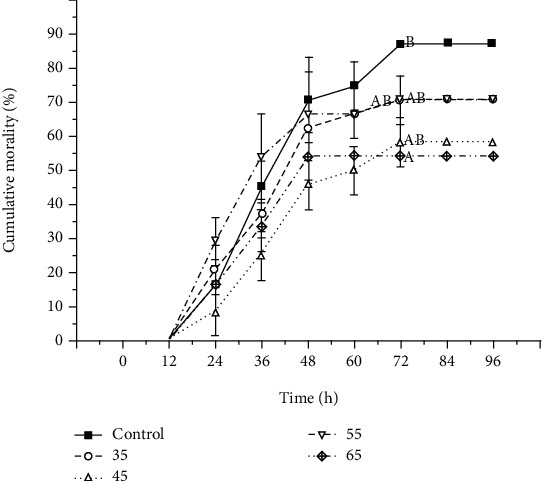
Effects of dietary proteolytic soybean meal on the resistance against *Vibrio alginolyticus* injection in *Litopenaeus vannamei*. Different superscript letters indicate significant differences (*P* < 0.05).

Monitoring the mortality of shrimp postpathogen challenge with a high dose of *V. alginolyticus* is shown in Figure 1. Dietary application of PSM significantly (*P* < 0.05) decreased the cumulative mortality of shrimp infected with *V. alginolyticus* after 48 h. Additionally, shrimp fed the diet containing 65 g/kg PSM showed a significantly lower cumulative mortality at 72 h when compared to the control.

### 3.6. Immune-Related Gene Expression

**Figure 2:**
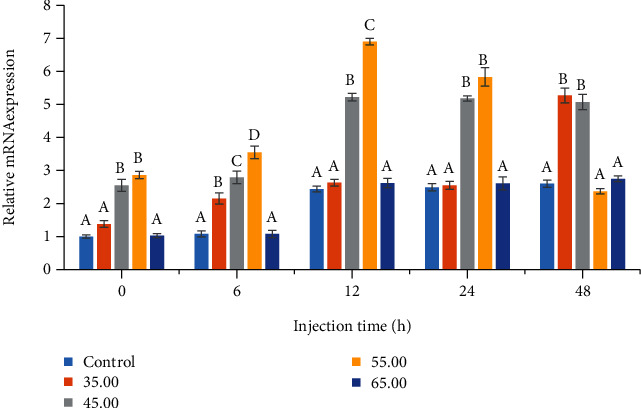
Effect of dietary proteolytic soybean meal on relative expression of Toll-like receptor 2 mRNA in the gill of *Litopenaeus vannamei* postchallenged to *Vibrio alginolyticus* at different times. Different superscript letters indicate significant differences (*P* < 0.05).

**Figure 3:**
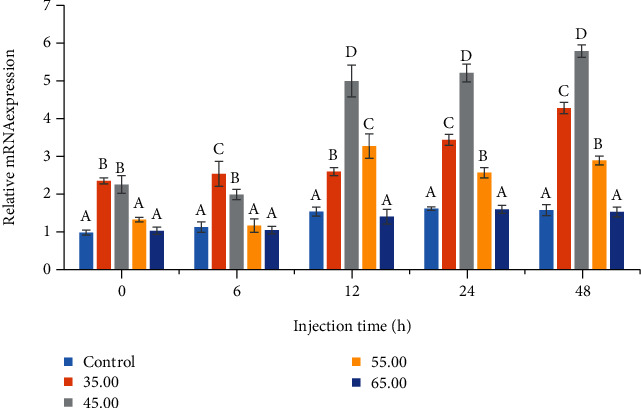
Effect of dietary proteolytic soybean meal on relative expression of immune deficiency (IMD) mRNA in the gill of *Litopenaeus vannamei* postchallenged to *Vibrio alginolyticus* at different times. Different superscript letters indicate significant differences (*P* < 0.05).

The expressions of genes related to Toll-like receptor 2 and immune deficiency (IMD) in the low-dose *V. alginolyticus*-infected *L. vannamei* are shown in Figures 2 and 3. Toll-like receptor 2 and IMD genes were remarkably (*P* < 0.05) upregulated in gill tissues from shrimp fed with PSM-supplemented diet compared to the control. Relative expressions of Toll-like receptor 2 mRNA reached maximum values at 12 h postinfection in 45 and 55 g/kg dietary PSM treatments. The most significantly relative expressions of IMD mRNA compared to the others were given by 45 g/kg PSM-supplemented treatment after 12 h postinfection of *V. alginolyticus.* Furthermore, there were no significant differences between the control and 65 g/kg level treatments on the expressions of these two genes.

## 4. Discussion

Formulated feed plays a vital role in the current aquaculture industry, consisting of 40-50% of total production costs [19]. Fish meal insufficiency has been recognized as a severe problem for the aquafeed industry. A significant byproduct of soybean oil production, soybean meal, contains many high-quality proteins rich in essential amino acids (EAA) for aquatic animals [8]. However, high levels of plant protein cannot be effectively utilized by shrimp without negative effects [15]. Their practical drawbacks are associated with EAA limitations (including lysine, methionine, and threonine) and antinutritional factors (ANFs) (such as protease inhibitors, lectins, and phytic acid). Moreover, soybean meal also contains high amounts of nonstarch polysaccharides. The presence of these ANFs and carbohydrate fractions in soybean meal could negative influence nutrient utilization, growth performance, and immune response of aquatic animals [10]. The fermentation process could improve the availability of vitamins, protein solubility, amino acid patterns, and palatability of soybean meal to overcome the limitations of reducing dietary fishmeal [5]. Furthermore, proteolysis has been used to generate various protein hydrolysates that could reduce the ANFs and enhance the peptide content with desired health functionalities [20].

The results in the current study clearly showed that the partial replacement of soybean meal with PSM significantly improved shrimp growth. In general, high growth rates require an abundant supply of dietary amino acids and small peptides for anabolic as well as energetic purposes [10]. The free amino acid, dipeptides, and tripeptides from proteolysis protein are readily absorbed by enterocytes and digested in the organism at a higher efficiency than proteins [4]. Therefore, supplementation with prehydrolyzed proteins in the diets is expected to improve protein availability and absorption rate, in addition to enhancing the growth of aquatic animals. The PSM used in the current study has substantially high levels of small peptides (molecular weight less than 1000 Da contains more than 73%). The increased growth in the current study, which was correlated with improved PER and PDR, was likely due to a relatively higher absorption efficiency of small peptides. However, the increased inclusion of PSM over 35 g/kg did not result in a more pronounced growth performance in the current study. A similar pattern was also found on FCR and PER. *L. vannamei* seems to present a limit on the growth response to protein hydrolysates related to the presence of large amounts of small peptides [21]. Hernández et al. [22] reported that improvements in growth performance were also observed in *L. vannamei* with a lower concentration of protein hydrolysates that were used in diets. These could be explained by the relative absorption efficiency of small peptide reduced by the limited amount of small peptide transporter as the dietary dose increased.

Processing soybean meal into PSM could remove lectins and trypsin inhibitors [23], which could also contribute to the higher growth in the PSM inclusive treatments. In the digestive glands of shrimp, trypsin activity is responsible for splitting protein down into smaller peptides, facilitating absorption [24]. Studies on shrimp fed with PSM have generally shown an increase in trypsin activity in the hepatopancreas, which agree with the current study [21]. Further, proteolysis can eliminate or reduce the content of many nonstarch polysaccharides, such as galactose, stachyose, and raffinose, and thus could improve the activity of amylase, as observed in the current study [25].

In addition to positively influencing protein turnover and growth, the bioactive peptides contents found in protein hydrolysates could improve the immune response of aquatic animal and their resistance to infectious diseases via the antimicrobial, antioxidant, and immunomodulatory activities [20, 26]. Malondialdehyde (MDA) is a secondary oxidation product of polyunsaturated fatty acids. Superoxide dismutase (SOD) can reduce O_2_^−^ accumulation by catalyzing superoxide anions via disproportionation reactions [16]. Thus, assaying of MDA and SOD is commonly used to indicate oxidative stress in tissues. Hepatopancreatic oxidative stress of white shrimp could be induced by the inclusion of soybean meal in the diet by exhibiting a higher MDA activity than do shrimp fed soybean meal free diet [27]. The hydrolytic processes could effectively reduce the effects of phytic acid in soybean meal, which has been demonstrated to interfere with the utilization of microelements [28]. Our results indicated that shrimp fed with PSM showed higher SOD and lower MDA activities than shrimp fed with conventional soybean meal. This finding supports that proteolysis can reduce the adverse effects of soybean on the oxidative status of white shrimp.

Shrimp depend entirely on the innate immune system to destroy invasive pathogens [16]. Lysozyme is an essential indicator of non-specific immunity for crustaceans [29]. Lysozyme activity in the serum showed a similar pattern to the SOD in the current study. The Toll and IMD are the most important signaling pathways that regulate the nonspecific immunity of shrimp [30]. An increase in the transcription of invasion signaling pathways effectors could directly reflect a sufficient and adequate immune response to pathogen invasion [31]. The relative expression levels of Toll-like receptor 2 and IMD mRNA in PSM inclusive treatments were upregulated in shrimp challenged with *V. alginolyticus*. However, the maximum expression levels of IMD and Toll-like receptor 2 mRNA were not simultaneous, mainly based on the activation of signaling pathways from an inactive form to an active form. The maximum relative mRNA expression levels of these two genes increased with the proper increment of supplemental PSM imply the positive and dose-related characteristics.

*V. alginolyticus* is a conditional pathogen of shrimp: the pathogen of this bacterium is associated with the presence of various virulence factors and the synergistic regulation of the expression of these factors by environmental stimuli [32]. The current study showed that shrimp fed with PSM were less susceptible to *V. alginolyticus* infection, especially for the 65 g/kg supplemental level treatment. These results were consistent with the change in the serum lysozyme and antioxidant capacity parameters.

In conclusion, the current study proved that dietary appropriate dose replacement of soybean meal with PSM in a low-fish-meal diet could result in better growth and immune status of *L. vannamei*. Notably, excessive supplementation did not produce further significant effects in the current study.

## Figures and Tables

**Table 1:** Composition of the experimental diets (g/kg dry matter basis).

Ingredients	Treatments				
	Control	35	45	55	65
Brown fish meal^a^	50	50	50	50	50
Corn protein powder^a^	80	80	80	80	80
Soybean meal^a^	305	270	260	250	240
Peanut meal^a^	80	80	80	80	80
Spray-dried blood powder^a^	35	35	35	35	35
Beer yeast cell^a^	40	40	40	40	40
Meat meal^a^	40	40	40	40	40
Methionine^a^	1	1	1	1	1
Flour^a^	235.7	235.7	235.7	235.7	235.7
[Ca(H_2_PO_4_)_2_]^a^	20	20	20	20	20
Soybean lecithin^a^	20	20	20	20	20
Fish oil^a^	35	35	35	35	35
Squid paste^a^	30	30	30	30	30
Mineral premix^b^	15	15	15	15	15
Vitamin premix^c^	10	10	10	10	10
Choline chloride^a^	3	3	3	3	3
VC phosphate^a^	0.3	0.3	0.3	0.3	0.3
Proteolytic soybean meal^d^	0	35	45	55	65
Total	1000	1000	1000	1000	1000
Proximate compositions (%)					
Moisture	11.84	12.37	12.12	12.29	12.09
Ash	7.73	7.36	7.53	7.31	7.18
Crude protein	39.55	39.28	39.45	39.14	39.59
Total lipid	12.40	12.54	12.76	12.70	12.19

^a^Guangdong Yuehai Feed Co., Ltd. ^b^Per kg mineral premix contains: 0.8 g Co, 0.02 g Se, 3 g Cu, 10 g Zn, 3.8 g Mn, 1 g Fe, 12 g Mg, 90 g K, and 10.5 g Ca. ^c^Per kg vitamin premix contains: 8 million IU of vitamin A, 5 g thiamine-HCl, 15 g riboflavin, 2 million IU of cholecalciferol, 50 g DL-*α*-tocopherol, 8 g pyridoxine-HCl, 10 g menadione, 0.02 g cyanocobalamin, 40 g nicotinamide, 25 g Ca-pantothenate, 2.5 g folic acid, 0.08 g biotin, and 100 g inositol. ^d^Taizhou Fuyi Biological Co., Ltd.

**Table 2:** Real-time quantitative PCR primers for immune-related genes and *β-actin* of white shrimp (*Litopenaeus vannamei*).

Primer	Nucleotide sequence (5′-3′)	GenBank reference
Toll-like receptor 2	F: TGGTGCTTTCGTCAAACTTC	DQ923424
	R: AACCTGGCCATACACAATGA	
IMD	F: ATCGAGGAACGAGACAAGGT	FJ592176
	R: CGTACACTCGGTCGACATTC	
*β*-Actin	F: CGCGACCTCACAGACTACCT	AF300705
	R: CTCGTAGGACTTCTCCAGCG	

**Table 3:** Growth performances and feed utilization of *Litopenaeus vannamei* fed with experimental diets.^1^.

Parameters^2^	Treatments				
	Control	35	45	55	65
FBW	13.65 ± 0.21^a^	14.31 ± 0.40^ab^	14.65 ± 0.49^b^	14.64 ± 0.68^b^	14.93 ± 0.37^b^
SGR	6.09 ± 0.03^a^	6.17 ± 0.05^ab^	6.22 ± 0.06^b^	6.22 ± 0.08^b^	6.25 ± 0.04^b^
Survival	98.00 ± 2.00	98.67 ± 1.16	97.33 ± 1.16	98.67 ± 1.16	96.67 ± 1.16
FCR	1.53 ± 0.03^b^	1.45 ± 0.03^a^	1.42 ± 0.04^a^	1.41 ± 0.05^a^	1.43 ± 0.04^a^
PER	1.64 ± 0.04^a^	1.76 ± 0.03^b^	1.79 ± 0.06^b^	1.82 ± 0.07^b^	1.77 ± 0.05^b^
PDR	31.41 ± 0.69^a^	35.99 ± 0.67^b^	36.40 ± 1.12^b^	37.69 ± 1.37^b^	37.03 ± 1.00^b^

^1^Data were expressed as means ± S.E.M. from triplicate groups. Different superscript letters indicate significant differences (*P* < 0.05). ^2^FBW (g): final body weight; SGR (%/day): specific growth ratio; FCR: feed conversion ratio; PER (%): protein efficiency ratio; PDR (%): protein deposition ratio.

**Table 4:** Biochemical analysis (% wet weight basis) of *Litopenaeus vannamei* muscle fed with experimental diets.^1^.

Parameters	Treatments				
	Control	35	45	55	65
Moisture	77.02 ± 1.49	76.65 ± 0.21	76.71 ± 1.97	76.50 ± 0.79	76.35 ± 0.49
Ash	1.40 ± 0.03	1.38 ± 0.09	1.37 ± 0.02	1.35 ± 0.13	1.34 ± 0.06
Crude protein	19.95 ± 0.13^a^	20.35 ± 0.22^ab^	20.30 ± 0.09^ab^	20.79 ± 0.19^b^	20.25 ± 0.15^ab^
Total lipid	1.18 ± 0.02	1.21 ± 0.02	1.23 ± 0.01	1.27 ± 0.04	1.26 ± 0.03

^1^Data were expressed as means ± S.E.M. from triplicate groups. Different superscript letters indicate significant differences (*P* < 0.05).

**Table 5:** Digestive enzyme activities of *Litopenaeus vannamei* fed with experimental diets^1^ (U/mg protein).

Parameters	Treatments				
	Control	35	45	55	65
Amylase	14.44 ± 0.95^a^	16.89 ± 1.51^ab^	17.54 ± 1.36^b^	16.70 ± 1.08^ab^	17.47 ± 1.15^b^
Lipase	59.54 ± 5.29^a^	71.75 ± 5.50^b^	68.01 ± 4.04^ab^	66.15 ± 7.53^ab^	69.21 ± 6.17^ab^
Protease	171.83 ± 0.96^a^	181.90 ± 1.85^b^	181.81 ± 7.50^b^	187.55 ± 6.43^b^	185.09 ± 2.40^b^

^1^Data were expressed as means ± S.E.M. from triplicate groups. Different superscript letters indicate significant differences (*P* < 0.05).

**Table 6:** Effects of dietary proteolytic soybean meal on serum lysozyme and antioxidant capacity of *Litopenaeus vannamei*.^1^.

Parameters	Treatments				
	Control	35	45	55	65
Lysozyme (U/mL)	40.16 ± 18.4^a^	100.40 ± 13.91^b^	104.42 ± 13.91^b^	112.45 ± 13.91^b^	120.48 ± 12.05^b^
SOD (U/mL)	374.22 ± 6.41^a^	391.87 ± 2.69^b^	398.33 ± 3.95^b^	395.75 ± 8.79^b^	402.64 ± 10.03^b^
MDA (nmol/mL)	69.26 ± 3.53^b^	65.19 ± 3.53^ab^	65.19 ± 9.33^ab^	63.15 ± 3.53^ab^	57.04 ± 3.53^a^

^1^Data were expressed as means ± S.E.M. from triplicate groups. Different superscript letters indicate significant differences (*P* < 0.05).

## Data Availability

The authors declare that the data supporting the findings of this study are available within the article.
